# Association of Medicaid Expansion With Emergency Department Visits by Medical Urgency

**DOI:** 10.1001/jamanetworkopen.2022.16913

**Published:** 2022-06-14

**Authors:** Theodoros V. Giannouchos, Benjamin Ukert, Christina Andrews

**Affiliations:** 1 Department of Health Services Policy & Management, Arnold School of Public Health, University of South Carolina, Columbia; 2 Department of Health Policy & Management, School of Public Health, Texas A&M University, College Station

## Abstract

**Importance:**

Relatively little is known about the association of the Medicaid eligibility expansion under the Patient Protection and Affordable Care Act with emergency department (ED) visits categorized by medical urgency.

**Objective:**

To estimate the association between state Medicaid expansions and ED visits by the urgency of presenting conditions.

**Design, Setting, and Participants:**

This cross-sectional study used the Healthcare Cost and Utilization Project State Emergency Department Databases from January 2011 to December 2017 for 2 states that expanded Medicaid in 2014 (New York and Massachusetts) and 2 states that did not (Florida and Georgia). Difference-in-differences regression models were used to estimate the changes in ED visits overall and further stratified by the urgency of the conditions using an updated version of the New York University ED algorithm between the states that expanded Medicaid and those that did not, before and after the expansion. Data were analyzed between June 7 and December 12, 2021.

**Exposure:**

State-level Medicaid eligibility expansion.

**Main Outcomes and Measures:**

Emergency department visits per 1000 population overall and stratified by medical urgency of the conditions.

**Results:**

In total, 80.6 million ED visits by 26.0 million individuals were analyzed. Emergency department visits were concentrated among women (59.3%), non-Hispanic Black individuals (28.3%), non-Hispanic White individuals (47.8%), and those aged 18 to 34 years (47.5%) and 35 to 44 years (20.4%). The rates of ED visits increased by a mean of 2.4 visits in nonexpansion states and decreased by a mean of 2.2 visits in expansion states after 2014, resulting in a significant regression-adjusted decrease of 4.7 visits per 1000 population (95% CI, −7.7 to −1.5; *P* = .003) in expansion states. Most of this decrease was associated with decreases in ED visits by conditions classified as not emergent (−1.5 visits; 95% CI, −2.4 to −0.7; *P* < .001), primary care treatable (−1.1 visits; 95% CI, −1.6 to −0.5; *P* < .001), and potentially preventable (−0.3 visits; 95% CI, −0.5 to −0.1; *P* = .02). No significant changes were observed for ED visits related to injuries and conditions classified as not preventable (−1.4; 95% CI, −3.1 to 0.3; *P* = .10), as well as for substance use and mental health disorders (0.0; 95% CI, −0.2 to 0.2; *P* = .94).

**Conclusions and Relevance:**

The findings of this study suggest that Medicaid expansion was associated with decreases in ED visits, for which decreases in ED visits for less medically emergent ED conditions may have been a factor.

## Key Points

**Question:**

Is Medicaid eligibility expansion associated with changes in emergency department (ED) visits based on the medical urgency of the conditions?

**Findings:**

In this cross-sectional study of 80.6 million ED visits across 4 US states, ED visits per 1000 population decreased for states that expanded Medicaid compared with states that did not. This decrease was associated with decreases in ED visits for less-emergent or nonemergent conditions.

**Meaning:**

The findings of this study suggest that expanding Medicaid might reduce ED visits for conditions that could be treated in outpatient settings.

## Teaser

This cross-sectional study examines changes in the use of emergency department services by the urgency of the condition between states that did vs those that did not expand Medicaid under the Affordable Care Act.

## Introduction

Emergency departments (EDs) are a vital component of the US health care system, with more than 140 million visits in 2018 incurring a total cost of approximately $75 billion.^1^ Emergency department visits are expected to increase even further in the years ahead, by a projected 12% by 2030.^2,3^ Although EDs treat patients with acute and unexpected health care conditions, they often serve as a safety net for individuals who are unable to access other health care settings. The use of EDs for nonemergent and preventable medical conditions has been a long-standing challenge in the US health care system. Every year, it is estimated that one-third of all ED visits occur for care that is treatable in primary care settings and preventable.^4,5,6^ At an average cost that is many times higher compared with treatment provided at a physician’s office or an urgent care center, some of these visits suggest inefficient allocation of resources and present a cost-saving opportunity.^7^

Limited or lack of insurance coverage is a key factor for many ED visits. Uninsured individuals often use the ED as a place to receive routine health care services and are also more likely to experience a health care crisis requiring emergent care owing to the lack of access to outpatient health services and preventive care.^8^ Social determinants of health among low-income beneficiaries that perpetuate difficulties in receiving routine health care services could also result in increased ED use for similar reasons.^9,10,11^

The expansion of Medicaid eligibility afforded by the Patient Protection and Affordable Care Act of 2010 (ACA) presented a unique opportunity to address this challenge by providing millions of US residents with health insurance coverage that could improve access to routine health care and preventive services. Since 2014, 35 US states have adopted the Medicaid eligibility expansion option, resulting in health insurance uptake for more than 20 million US residents.^12,13^ Larger gains in enrollment were documented in states that decided to expand Medicaid eligibility to adults less than age 65 years with incomes up to 138% of the federal poverty level.^14,15,16,17^ The implementation of the Medicaid expansion has also been associated with increased use of preventive services, access to primary care clinicians, affordability, and quality of care.^13,16,17,18,19,20,21,22,23^

However, the evidence on the association between Medicaid expansion and ED use is inconclusive.^18,24^ Limited or no cost-sharing for preventive and primary care services could lead to a shift of care from the ED setting to outpatient care, after obtaining new or more generous health care coverage. Recent studies reported that Medicaid expansion was associated with decreases in ED visits among individuals who previously reported barriers to outpatient care and those related to opioid use.^25,26^ However, the expansion could have also increased ED use by eliminating or reducing cost barriers to receiving care in the ED.^18,27,28,29,30,31^ A recent study by Garthwaite and colleagues^32^ noted that Medicaid expansion was associated with increases in ED visits for deferrable medical conditions, defined as visits for which the patient has some discretion regarding when and where to seek care.

These results highlight a complex relationship between health insurance and ED use.^33^ However, analyses examining only total ED visits may conceal important changes in the composition of the ED visits by medical urgency. A recent study analyzing ED visits after Medicaid expansion highlighted the importance of further studies that examine nonemergent ED use.^24^ Hence, there remains a need to categorize ED visits by medical urgency because parameters that divert individuals with different conditions from use of EDs might vary.^34^

To address this gap in the literature, we estimated the association between the ACA Medicaid expansion and ED visits, stratified by medical urgency of the visits, using a validated algorithm, and compared 2 Medicaid-expansion states with 2 nonexpansion states.

## Methods

### Data Sources

Our main databases were the Healthcare Cost and Utilization Project State Emergency Department Databases from January 2011 to December 2017.^35^ These longitudinal administrative secondary databases include all-payer discharge information for nearly all outpatient (treat and release) ED visits across every general and acute care hospital within a state, similar to previous work.^31^ We focused on outpatient ED visits, which account for almost 90% of all ED visits, to identify encounters that can be classified as less emergent.^36^ We included data from 4 states (Florida, Georgia, Massachusetts, and New York), which account for almost one-fifth of the US population. Two of these states expanded Medicaid in 2014 (Massachusetts and New York), and the others did not. We included in-state residents aged 18 to 64 years who were covered by Medicaid, private plans or other local, state, or federal plans, or who were uninsured throughout the study period. Medicare enrollees were not included in the study given that individuals enrolled in the program were not directly affected by the Medicaid eligibility expansion and would be unlikely to transition from Medicare to Medicaid as a result of the policy change. We followed the Strengthening the Reporting of Observational Studies in Epidemiology (STROBE) reporting guideline for reporting observational studies. The study was determined to be not human subjects research by the Texas A&M University Institutional Review Board.

### Outcomes of Interest and Measurement

Our outcomes of interest were total ED visits and ED visits classified by medical urgency per 1000 population. We obtained publicly available annual state-level population counts for the corresponding age groups to generate ED visits per 1000 residents.^37^ We then used the updated version of the New York University ED algorithm to classify each ED visit by medical urgency, based on the primary diagnosis code from the *International Classification of Diseases, 9th Revision* (January 2011 to September 2015) or *International Statistical Classification of Diseases, 10th Revision* (October 2015 to December 2017).^6,38^ This algorithm assigns probabilities and classifies each ED visit into 1 or multiple probability-adjusted categories: emergent and not-preventable or avoidable (eg, chest pain, tachycardia); emergent but preventable or avoidable (eg, dehydration); emergent but primary care–treatable (eg, muscle strain); not emergent (eg, low back pain, headache); injury; and alcohol, drug use, and mental health–related issues. We averaged the algorithmically assigned probabilities and grouped ED visits into 5 categories to reflect conditions by medical urgency: (1) not preventable and injury-related, (2) emergent but potentially preventable, (3) emergent but primary care treatable, (4) not emergent, and (5) mental health and substance use disorders.^28^ We aggregated data at the state year-quarter level similar to previous work.^24,26^

### State Medicaid Expansion Definition

The primary exposure was state Medicaid expansion status. We obtained and identified the status of the decisions to expand Medicaid from publicly available resources.^39^ Massachusetts and New York adopted the ACA Medicaid expansion provision in January 2014; Georgia and Florida did not.

### Covariates

We also included publicly available, time-varying, state-level variables in our analyses that have been commonly associated with ED use as control variables (percentages of women; non-Hispanic Black, Hispanic, and non-Hispanic White individuals; age-group distributions; annual unemployment rate; and percentage of population under 200% of the federal poverty level).^37,40,41^ Race and ethnicity data were obtained from publicly available resources at the state-year level to control for state-level variation. The categorization is how this information was available on the publicly available resource.

### Statistical Analysis

We conducted a descriptive analysis for all 4 states and then stratified by expansion status. We then performed difference-in-differences regression analyses to estimate the association of the Medicaid expansion with ED visits that were weighted by each state’s population with state year-quarter as the unit of analysis.^31^ This approach enabled us to compare pre-ACA expansion vs post-ACA expansion outcomes in states that implemented the Medicaid expansion (treatment group) with states that did not (control group). Our adjusted regression analyses included all covariates. Robust SEs were used. We also conducted sensitivity analyses without state-level population weights to evaluate the sensitivity of the findings. Moreover, we evaluated the year-by-year difference-in-differences in ED visits by interacting the Medicaid expansion indicator of the states with each year separately. Because Massachusetts implemented a large health reform before the ACA to provide near-universal health insurance coverage, we conducted supplemental analyses by only comparing New York with Florida. These 2 states had similar rates of ED visits in the preexpansion period.

One critical assumption of the difference-in-differences model is that both the treatment and control groups exhibited parallel trends in the prepolicy implementation period.^42^ We examined trends in the pre-ACA expansion period (2011-2013) by conducting regressions across all outcomes with an interaction term between year-quarters and the expansion status dummy as the primary independent variable. The results showed little evidence of diverging trends and provided support of the parallel trends assumption (eTable 1 in the Supplement). Two-tailed tests were used, and statistical significance was considered at *P* < .05. We managed the data using SAS, version 9.4 (SAS Institute Inc) and all statistical analyses were performed between June 7 and December 12, 2021, using Stata, version 17.0 (StataCorp LLC).

## Results

### Characteristics of ED Visits

Our study included 80.6 million ED visits by 26.0 million individuals. Table 1 presents descriptive information for all states and stratified by Medicaid expansion status from 2011 to 2017. Overall, 59.3% of visits were by women and 40.7% by men, with 67.9% of the visits by those aged between 18 and 44 years (18-34 years, 47.5%; 35-44 years, 20.4%). With classification by race and ethnicity status, 28.3% of the visits were by non-Hispanic Black individuals, 15.8% by Hispanic individuals, 47.8% by non-Hispanic White individuals, and 8.1% by those of other racial and ethnic groups (ie, Asian/Pacific Islander, Native American, or other race and ethnicity). Across all states, about one-third (32.1%) of the ED visits were for injury-related or not-preventable conditions, 22.9% were classified as not emergent, 21.8% as primary care treatable, and 5.3% as potentially preventable. Visits for mental health and substance use disorders accounted for 4.8% of all visits but were relatively higher in expansion states compared with nonexpansion states (6.4% vs 3.3%; *P* < .001). The share of Medicaid-paid ED visits was disproportionately higher in expansion states compared with nonexpansion states (41.5% vs 25.4%; *P* < .001), and the opposite was observed for the uninsured population (11.2% vs 35.1%; *P* < .001).

**Table 1.** Descriptive Statistics for All States and Stratified by Medicaid Expansion Status From 2011 to 2017

Variable	All states	Nonexpansion states	Expansion states
Before Medicaid expansion (2011-2013)			
Total No. of quarterly visits per 1000,mean (SD)	52.1 (3.3)	53.9 (3.6)	50.5 (2.0)
After Medicaid expansion (2014-2017)			
Total No. of quarterly visits per 1000, mean (SD)	52.3 (4.7)	56.3 (2.0)	48.3 (2.9)
Total No. of ED visits	80 565 423	44 830 589	35 734 834
Total No. quarterly visits per 1000, mean (SD)	52.3 (4.2)	54.6 (3.0)	49.8 (2.7)
Classification by medical urgency			
Injuries/emergent–not preventable			
No. of visits per 1000, mean (SD)	16.8 (1.6)	17.3 (1.3)	16.2 (1.7)
Share of total quarterly visits per 1000, %	32.1	31.7	32.5
Emergent–primary care treatable			
No. of visits per 1000, mean (SD)	11.4 (1.5)	12.5 (0.6)	10.1 (0.6)
Share of total quarterly visits per 1000, %	21.8	22.9	20.3
Emergent–potentially preventable			
No. of visits per 1000, mean (SD)	2.8 (0.4)	2.9 (0.3)	2.6 (0.2)
Share of total quarterly visits per 1000, %	5.3	5.3	5.2
Not emergent			
No. of visits per 1000, mean (SD)	12.0 (1.5)	12.7 (1.0)	11.1 (1.2)
Share of total quarterly visits per 1000, %	22.9	23.3	22.3
Mental health and substance use disorders			
No. of visits per 1000, mean (SD)	2.5 (0.8)	1.8 (0.2)	3.2 (0.3)
Share of total quarterly visits per 1000, %	4.8	3.3	6.4
Unclassified			
No. of visits per 1000, mean (SD)	6.8 (1.5)	7.4 (1.5)	6.6 (1.2)
Share of total quarterly visits per 1000, %	13.1	13.5	13.3
Characteristics of ED users			
Sex, mean (SD), share, %			
Male	40.7 (3.2)	38.2 (0.5)	43.7 (1.4)
Female	59.3 (3.3)	61.8 (0.5)	56.3 (1.5)
Race and ethnicity, mean (SD), share, %			
Hispanic	15.8 (5.7)	13.7 (6.7)	18.1 (1.4)
Non-Hispanic			
Black	28.3 (11.6)	33.2 (9.0)	22.8 (7.0)
White	47.8 (9.9)	48.6 (5.3)	46.9 (12.6)
Other^a^	8.1 (5.3)	4.5 (4.1)	12.2 (4.9)
Age group range, y, mean (SD), share, %			
18-34	47.5 (4.1)	48.8 (1.0)	46.0 (5.3)
35-44	20.4 (0.7)	20.8 (0.6)	19.9 (0.4)
45-54	18.9 (0.7)	18.3 (0.2)	19.5 (0.3)
55-64	13.2 (4.1)	12.1 (0.9)	14.6 (5.5)
Health insurance coverage, mean (SD), share, %			
Medicaid	33.5 (9.4)	25.4 (4.5)	41.5 (4.9)
Private	35.3 (3.9)	32.5 (2.8)	38.0 (2.7)
Uninsured	23.1 (13.2)	35.1 (4.8)	11.2 (6.2)
Other/unknown	8.1 (3.6)	7.0 (1.7)	9.2 (4.5)

Abbreviation: ED, emergency department.

^a^ Asian/Pacific Islander, Native American, or other race and ethnicity.

### Trends in ED Visits

**Figure 1.**
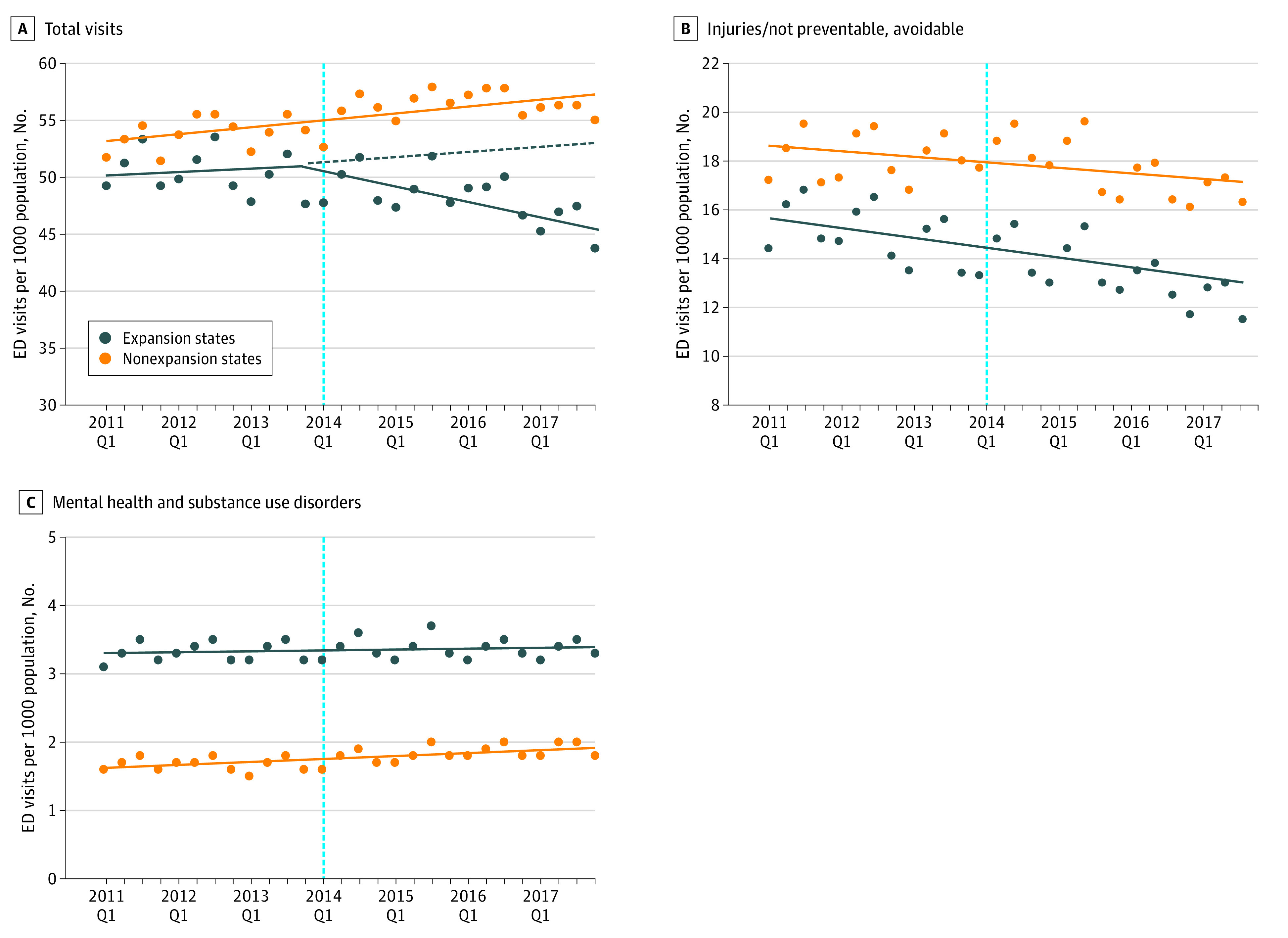
Emergency Department (ED) Total Visits per 1000 Population Overall and for Conditions Classified by the New York University Algorithm by Medicaid Expansion Status Total visits (A), injuries and not-preventable or avoidable visits (B), and visits related to mental health and substance use disorders (C). Q1 indicates quarter 1.

**Figure 2.**
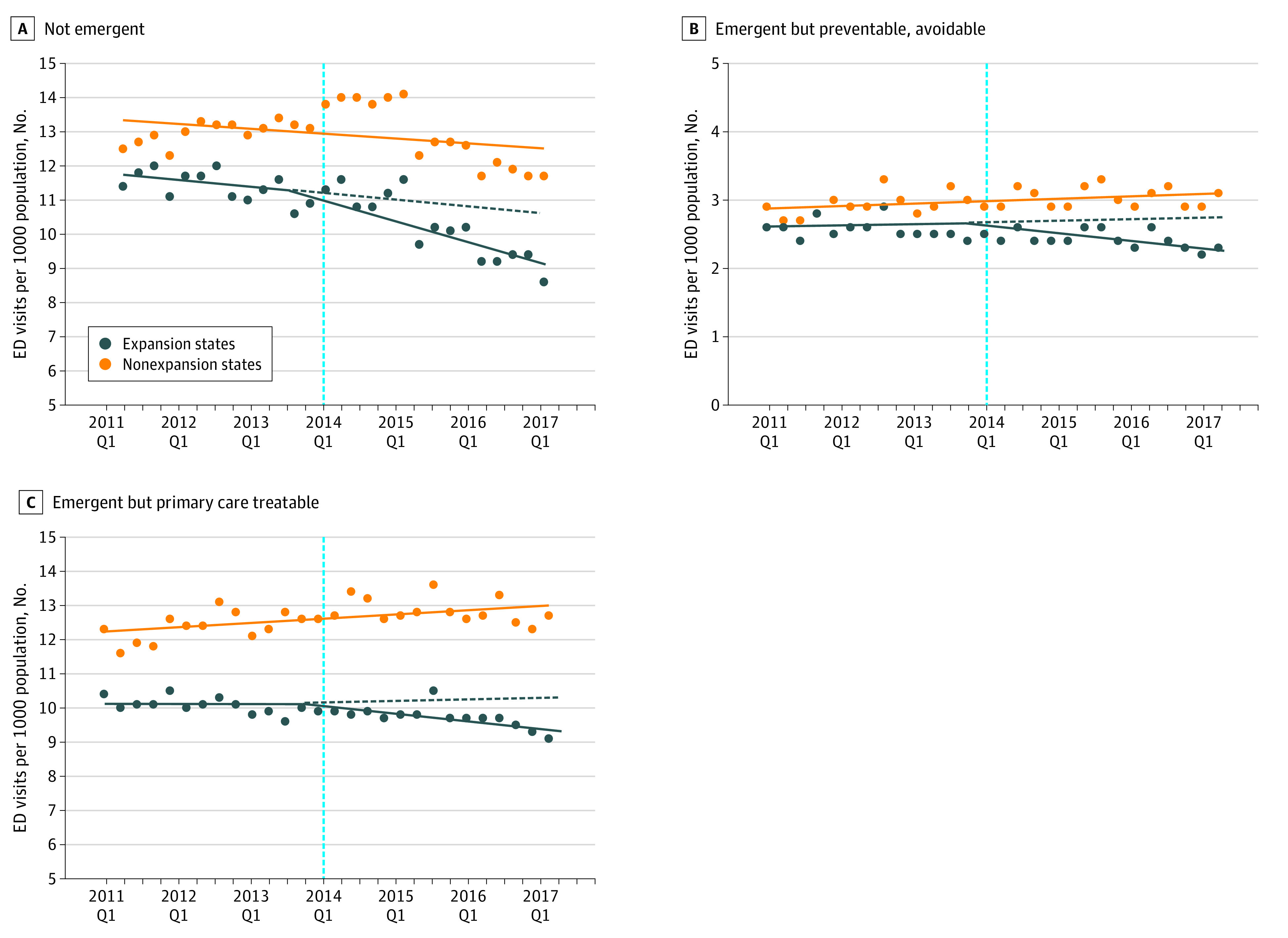
Emergency Department (ED) Visits per 1000 Population by Emergent Category Classified by the New York University Algorithm by Medicaid Expansion Status Nonemergent (A), emergent but preventable or avoidable (B), and emergent but primary care–treatable conditions (C). Q1 indicates quarter 1.

Overall, total ED visits per 1000 population remained similar in the pre- and post-ACA periods (52.1 vs 52.3 visits) while the total number of ED visits increased by approximately 1% per year on average (Figure 1; eFigure in the Supplement). Figure 1 presents the trends in total ED visits, injuries and not-preventable or avoidable issues, and mental health–related or substance use issues, and Figure 2 shows the trends by medical urgency per 1000 population from 2011 to 2017 in expansion and nonexpansion states. Across all outcomes, states had relatively similar ED visit trends before January 2014 (preexpansion period). Total ED visits per 1000 population decreased after 2014 in expansion states (4.3% mean relative decrease) and increased in nonexpansion states (4.5% mean relative increase). Emergency department visits for conditions classified as potentially preventable (6.1% mean relative decrease) and primary care treatable (3.2% mean relative decrease) decreased only in expansion states, and those for not-emergent conditions decreased in both expansion (10.3% mean relative decrease) and nonexpansion (0.7% mean relative decrease) states. Visits for injuries and not-preventable conditions decreased in both expansion and nonexpansion states (mean relative decrease 11.3% and 2.0%), and those related to mental health and substance use disorders followed relatively flat trends across expansion and nonexpansion states.

### Difference-in-Differences Analyses of ED Visits

Table 2 reports unadjusted and adjusted regression difference-in-differences results. Total ED visits per 1000 population increased by 2.4 visits in nonexpansion states and decreased by 2.2 visits in Medicaid expansion states after 2014 compared with the pre-ACA period. This change resulted in a significant regression-adjusted decrease of 4.7 ED visits per 1000 population (95% CI, −7.7 to −1.5; *P* = .003). Compared with nonexpansion states, the ACA was associated with decreases of 1.5 ED visits per 1000 population (95% CI, −2.4 to −0.7; *P* < .001) for not-emergent, 1.1 ED visits per 1000 population (95% CI, −1.6 to −0.5; *P* < .001) for primary care–treatable, and 0.3 ED visits per 1000 population (95% CI, −0.5 to −0.1; *P* = .02) for potentially preventable conditions in states that opted in the expansion of Medicaid. We did not observe any significant differences in ED visits for injuries or not-preventable conditions (−1.4; 95% CI, −3.1 to 0.3; *P* = .10), and visits related to mental health and substance disorders (0.0; 95% CI, −0.2 to 0.2; *P* = .94). The year-by-year trend difference-in-differences in the post–Medicaid expansion period showed that ED visits overall decreased each year as well as for less-emergent conditions (eTable 2 in the Supplement). Medicaid expansion was also associated with decreases in ED visits for injuries and not-preventable conditions, but only after the third year of the policy implementation (2016: −2.6; 95% CI, −4.3 to −0.8; *P* = .004; 2017: −3.3; 95% CI, −5.6 to −1.1; *P* = .004). In addition, the sensitivity analyses without state population size-adjusted weights and the sample comparing only New York with Florida yielded similar results (eTable 3 in the Supplement).

**Table 2.** Difference-in-Differences Regression Analyses: Total ED Visits per 1000 Population and Stratified by Medical Urgency^a^

No. of ED visits per 1000 population	Nonexpansion states	Expansion states	Difference	Difference-in-differences		*P* value
				Unadjusted	Adjusted (95% CI)^b^	
**Overall**						
Total						
Before Medicaid expansion	53.9	50.5	−3.4	−4.6	−4.7 (−7.7 to −1.5)	.003
After Medicaid expansion	56.3	48.3	−8.0			
Injuries, not preventable						
Before Medicaid expansion	17.6	17.2	−0.4	−1.2	−1.4 (−3.1 to 0.3)	.10
After Medicaid expansion	17.2	15.6	−1.6			
Not emergent						
Before Medicaid expansion	12.7	11.6	−1.1	−1.0	−1.5 (−2.4 to −0.7)	<.001
After Medicaid expansion	12.7	10.6	−2.1			
Primary care treatable						
Before Medicaid expansion	12.2	10.2	−2.0	−0.8	−1.1 (−1.6 to −0.5)	<.001
After Medicaid expansion	12.8	10.0	−2.8			
Potentially preventable						
Before Medicaid expansion	2.9	2.6	−0.3	−0.2	−0.3 (−0.5 to −0.1)	.02
After Medicaid expansion	3.0	2.5	−0.5			
Mental health and substance use disorders						
Before Medicaid expansion	1.6	3.2	1.6	−0.1	0.0 (−0.2 to 0.2)	.94
After Medicaid expansion	1.8	3.3	1.5			

Abbreviation: ED, emergency department.

^a^ The analysis contained 112 state year-quarters, 28 for each state. Of those, 48 state year quarters correspond to the pre-Medicaid expansion years (2011-2013). Results show adjusted differences-in-differences weighted estimates for 2 expansion states (Massachusetts and New York) vs 2 nonexpansion states (Florida and Georgia). Classification of ED visits by medical urgency was conducted using the New York University ED algorithm.

^b^ Controlled for age, sex, race and ethnicity, poverty levels, and unemployment.

## Discussion

In this analysis of 80.6 million ED visits from 2011 to 2017 in 4 states, we found that Medicaid expansion was associated with a significant decrease of 4.7 ED visits per 1000 population in states that expanded Medicaid compared with states that did not, for which decreases in ED visits for less-emergent or not-emergent conditions may be a factor. We did not observe any major policy-related differences in ED visits for mental health and substance use disorders, injuries, and nonpreventable conditions overall.

Our results are consistent with previous work that found significant decreases in ED visits following the ACA implementation in states that expanded Medicaid.^17,25,42,43^ Our study expands the evidence using 4 postexpansion years and focuses on populous states that might more specifically represent the national trends in ED visits.^44^ Previous work with diverging results generally assessed changes in ED visits after 1 or 2 years postexpansion, focused their analysis on different states and populations, or reviewed outcomes using different definitions of not-emergent ED visits.^25,31,32^

The observed decreases in ED visits concurrently occurred with increases in availability and access to primary care, and more primary care professionals accepting patients with Medicaid coverage to higher Medicaid payment rates.^17,25,43,45,46,47^ Furthermore, decreases in Medicaid expansion states were concentrated in conditions that were less-emergent or not medically emergent, suggesting that access to preventive services could have substituted ED visits, improved health, and stabilized health conditions, which rendered ED use not necessary.^25,48,49,50,51^ Before implementation of the ACA, EDs were the only access point for many individuals owing to financial difficulties in identifying cost-effective avenues of care.^52^

However, ED visits for potentially preventable, primary care–treatable, and not-emergent conditions made up more than 40% of all ED visits even after states expanded Medicaid.^4,5^ Although expanding health insurance coverage may be important, it does not guarantee access to medical care. Time and access barriers to outpatient care, such as appointment availability, inconvenient office hours, underinsurance, infrastructure barriers (eg, waiting times and lack of diagnostic capabilities of primary care offices), and prevailing social needs (eg, housing and food instability), are commonly cited reasons for nonemergent presentations to EDs even among insured individuals.^4,5,9,10,25,33^

The high rates of ED visits for not-emergent conditions also raise concerns about the quality of care that some patients receive in primary care and outpatient settings. Dissatisfaction with primary care professionals, language barriers and unclear instructions, physician referrals to the ED, convenient operating hours, and the need for a second opinion are additional factors that predispose ED use.^4,10^ Furthermore, many individuals are not equipped to accurately perceive the severity of their condition and might overestimate the need for emergency care, particularly after regular office hours or when outpatient care is not available. In addition, lack of knowledge about viable alternatives and limited information about outpatient clinicians’ resources may further predispose nonemergent ED presentations.^4,25,53,54^

We also observed a small increase in ED visits related to mental health and substance use disorders across both expansion and nonexpansion states.^23^ Our data fall within the years of the second wave of the opioid epidemic in the US, when ED visits for substance use disorders increased by more than 30%.^55,56^ Factors such as fear of stigmatization, service availability, and health plan benefit constraints are additional barriers and considerations that these individuals face before entering treatments.^57,58,59^

Beyond the ACA expansion, we also noted an annual increase in the total number of ED visits of approximately 1%, almost twice the rate of annual population growth.^2^ The increasing demand for ED services warrants new and revised treatment protocols and models of care among emergency medicine professionals. Policies that might contain ED use include higher outpatient Medicaid reimbursement rates for behavioral and substance use treatment services and promoting the use of telemedicine.^16,31,46,55,60^ In addition, targeted outreach efforts to increase health plan enrollment may yield long-term benefits, as more than half of the uninsured population is eligible for Medicaid or subsidized coverage.^61^

However, addressing only medical and health care system factors will not reverse the social and economic circumstances that exacerbate access to care and chronic health problems. Synergies between medical and social needs demonstrated that coordination of medical, behavioral, and social services can improve health outcomes and contain ED use.^62^ Investments in social welfare services, data sharing, and integration of primary care, housing, food, and psychosocial services are needed to improve health and allocate resources more effectively. In addition, investments in health care professional education to promote patient engagement in the development of personal care plans with need-oriented goal setting are necessary to enable the health care system to evolve from medical care centered to patient centered.

### Limitations

Our study has limitations. First, we used data from only 4 states that exhibit similar ED visit rates with national trends, but findings may differ by state, as previous work using similar methods but different states found increases in ED visits.^31^ However, the difference in the results might be associated with either the use of more states or the use of data only including the first year of the Medicaid expansion. In addition, although the State Emergency Department Databases data represent almost 90% of all ED visits, we did not include data on ED visits that resulted in hospital admissions; thus, findings might not be generalizable to all ED visits. Second, there are differences in the proportion of each state’s population that became eligible for Medicaid in 2014.^24^ Massachusetts implemented a partial Medicaid expansion in 2006 and consequently added far fewer residents to Medicaid in 2014. However, this limitation is likely to result in an underestimation of the association of Medicaid expansion with observed trends rather than an overestimation. Third, the transition from *International Classification of Diseases, 9th Revision* to *International Statistical Classification of Diseases, 10th Revision* occurred during the study period, resulting in a shift in the codes used to identify visit types. Nonetheless, sensitivity tests conducted to check for bias in visit types, including unclassified visits, did not result in any significant change in our study findings. Fourth, owing to the nature of the New York University algorithm, it is possible that some categories were nonmutually exclusive and, thus, some conditions could be assigned to more than one group, which could bias our estimates. Fifth, the discharge diagnoses based on the retrospective assignment of probabilities by the New York University algorithm do not capture the patients’ perception of risk at the time of the episode and ED visit. Sixth, the retrospective design may be subject to potential unobserved confounders that could bias our results.

## Conclusions

The findings of this study suggest that the Medicaid expansion that occurred with implementation of the ACA was associated with significant reductions in ED visits in states that expanded Medicaid, for which decreases in ED visits for less medically emergent conditions, some of which could potentially be treated in other settings, may be a factor. However, ED visits for potentially preventable and primary care–treatable conditions continued to account for a large share of all ED visits, even in expansion states. As policy makers debate the future of the ACA and public support for a single-payer national health plan increases, our findings provide further data suggesting that investing in health insurance can reduce ED use for nonemergent medical conditions.

## Notes

Supplement. **eFigure.** Total Annual Number of Emergency Department Visits Across States From 2011 to 2017 **eTable 1.** Indirect Test of the Parallel Trends Assumption in the Pre-ACA Implementation Period **eTable 2.** Difference-in-Differences Regression Analyses Estimating Postexpansion Years Separately: Total ED Visits per 1000 Population and Stratified by Medical Urgency **eTable 3.** Difference-in-Differences Regression Analyses: Total ED Visits per 1000 Population and Stratified by Medical Urgency Between Florida and New York

## References

[1] Moore BJ, Liang L. Costs of emergency department visits in the United States, 2017. HCUP Statistical Brief #268. Agency for Healthcare Research and Quality. December 2020. Accessed October 14, 2021. http://www.hcup-us.ahrq.gov/reports/statbriefs/sb268-ED-Costs-2017.pdf 33439600

[2] Marco CA, Courtney DM, Ling LJ, . The Emergency Medicine Physician Workforce: Projections for 2030. Ann Emerg Med. 2021;78(6):726-737. doi:10.1016/j.annemergmed.2021.05.029 34353653

[3] Agency for Healthcare Research and Quality. Healthcare Cost and Utilization Project (HCUP): Nationwide Emergency Department Sample (NEDS) 2009-2018 (as of January 27, 2021). Accessed October 14, 2021. https://hcup-us.ahrq.gov/faststats/NationalTrendsEDServlet

[4] Uscher-Pines L, Pines J, Kellermann A, Gillen E, Mehrotra A. Deciding to visit the emergency department for non-urgent conditions: a systematic review of the literature. Am J Manag Care. 2013;19(1):47-59. 23379744 PMC4156292

[5] Giannouchos TV, Kum HC, Gary JC, Morrisey MA, Ohsfeldt RL. The effect of expanded insurance coverage under the Affordable Care Act on emergency department utilization in New York. Am J Emerg Med. 2021;48:183-190. doi:10.1016/j.ajem.2021.04.076 33964693

[6] Johnston KJ, Allen L, Melanson TA, Pitts SR. “Patch” to the NYU emergency department visit algorithm. Health Serv Res. 2017;52(4):1264-1276. doi:10.1111/1475-6773.12638 28726238 PMC5517669

[7] UnitedHealth Group. 18 Million avoidable hospital emergency department visits add $32 billion in costs to the health care system each year. July 2019. Accessed October 15, 2021. https://www.unitedhealthgroup.com/viewer.html?file=/content/dam/UHG/PDF/2019/UHG-Avoidable-ED-Visits.pdf

[8] Gindi RM, Black LI, Cohen RA. Reasons for emergency room use among US adults aged 18–64: National Health Interview Survey, 2013 and 2014. National Center for Health Statistics Reports. February 8, 2016. Accessed October 14, 2021. https://www.cdc.gov/nchs/data/nhsr/nhsr090.pdf 26905514

[9] Allen EM, Call KT, Beebe TJ, McAlpine DD, Johnson PJ. Barriers to care and healthcare utilization among the publicly insured. Med Care. 2017;55(3):207-214. doi:10.1097/MLR.0000000000000644 27579910 PMC5309146

[10] Cheung PT, Wiler JL, Lowe RA, Ginde AA. National study of barriers to timely primary care and emergency department utilization among Medicaid beneficiaries. Ann Emerg Med. 2012;60(1):4-10.e2. doi:10.1016/j.annemergmed.2012.01.035 22418570

[11] Bodenheimer TS, Smith MD. Primary care: proposed solutions to the physician shortage without training more physicians. Health Aff (Millwood). 2013;32(11):1881-1886. doi:10.1377/hlthaff.2013.0234 24191075

[12] Frean M, Gruber J, Sommers BD. Premium subsidies, the mandate, and Medicaid expansion: coverage effects of the Affordable Care Act. J Health Econ. 2017;53:72-86. doi:10.1016/j.jhealeco.2017.02.004 28319791

[13] Sommers BD, Maylone B, Blendon RJ, Orav EJ, Epstein AM. Three-year impacts of the Affordable Care Act: improved medical care and health among low-income adults. Health Aff (Millwood). 2017;36(6):1119-1128. doi:10.1377/hlthaff.2017.0293 28515140

[14] Lallemand NC. Reducing waste in health care. Health Aff (Millwood). 2012;13:1-5. doi:10.1377/hpb20121213.959735

[15] Courtemanche Ch, Marton J, Ukert B, Yelowitz A, Zapata D. Early impacts of the Affordable Care Act on health insurance coverage in Medicaid expansion and non-expansion states. J Policy Anal Manage. 2017;36(1):178-210. doi:10.1002/pam.21961 27992151

[16] Mazurenko O, Balio CP, Agarwal R, Carroll AE, Menachemi N. The effects of Medicaid expansion under the ACA: a systematic review. Health Aff (Millwood). 2018;37(6):944-950. doi:10.1377/hlthaff.2017.1491 29863941

[17] Sommers BD, Blendon RJ, Orav EJ, Epstein AM. Changes in utilization and health among low-income adults after Medicaid expansion or expanded private insurance. JAMA Intern Med. 2016;176(10):1501-1509. doi:10.1001/jamainternmed.2016.4419 27532694

[18] Sommers BD, Gawande AA, Baicker K. Health insurance coverage and health—what the recent evidence tells us. N Engl J Med. 2017;377(6):586-593. doi:10.1056/NEJMsb1706645 28636831

[19] Griffith KN, Bor JH. Changes in health care access, behaviors, and self-reported health among low-income US adults through the fourth year of the Affordable Care Act. Med Care. 2020;58(6):574-578. doi:10.1097/MLR.0000000000001321 32221101 PMC8133296

[20] Pukurdpol P, Wiler JL, Hsia RY, Ginde AA. Association of Medicare and Medicaid insurance with increasing primary care–treatable emergency department visits in the United States. Acad Emerg Med. 2014;21(10):1135-1142. doi:10.1111/acem.12490 25308137 PMC7255778

[21] Wallace DJ, Donohue JM, Angus DC, . Association between state Medicaid expansion and emergency access to acute care hospitals in the United States. JAMA Netw Open. 2020;3(11):e2025815. doi:10.1001/jamanetworkopen.2020.25815 33196808 PMC7670316

[22] Lam MB, Phelan J, Orav EJ, Jha AK, Keating NL. Medicaid expansion and mortality among patients with breast, lung, and colorectal cancer. JAMA Netw Open. 2020;3(11):e2024366. doi:10.1001/jamanetworkopen.2020.24366 33151317 PMC7645694

[23] Patel MR, Tipirneni R, Kieffer EC, . Examination of changes in health status among Michigan Medicaid expansion enrollees from 2016 to 2017. JAMA Netw Open. 2020;3(7):e208776. doi:10.1001/jamanetworkopen.2020.8776 32648922 PMC7352154

[24] Zhao F, Nianogo RA. Medicaid expansion’s impact on emergency department use by state and payer. Value Health. Published online October 26, 2021. doi:10.1016/j.jval.2021.09.014 35365307

[25] Chou SC, Gondi S, Weiner SG, Schuur JD, Sommers BD. Medicaid expansion reduced emergency department visits by low-income adults due to barriers to outpatient care. Med Care. 2020;58(6):511-518. doi:10.1097/MLR.0000000000001305 32000172

[26] Wen H, Soni A, Hollingsworth A, . Association between Medicaid expansion and rates of opioid-related hospital use. JAMA Intern Med. 2020;180(5):753-759. doi:10.1001/jamainternmed.2020.0473 32202609 PMC7091455

[27] Finkelstein AN, Taubman SL, Allen HL, Wright BJ, Baicker K. Effect of Medicaid coverage on ED use—further evidence from Oregon’s experiment. N Engl J Med. 2016;375(16):1505-1507. doi:10.1056/NEJMp1609533 27797307

[28] Taubman SL, Allen HL, Wright BJ, Baicker K, Finkelstein AN. Medicaid increases emergency-department use: evidence from Oregon’s health insurance experiment. Science. 2014;343(6168):263-268. doi:10.1126/science.1246183 24385603 PMC3955206

[29] Buchmueller TC, Grumbach K, Kronick R, Kahn JG. The effect of health insurance on medical care utilization and implications for insurance expansion: a review of the literature. Med Care Res Rev. 2005;62(1):3-30. doi:10.1177/1077558704271718 15643027

[30] Cunningham P, Sheng Y. Trends in preventable inpatient and emergency department utilization in California between 2012 and 2015. Med Care. 2018;56(6):544-550. doi:10.1097/MLR.0000000000000851 29298175

[31] Nikpay S, Freedman S, Levy H, Buchmueller T. Effect of the Affordable Care Act Medicaid expansion on emergency department visits: evidence from state-level emergency department databases. Ann Emerg Med. 2017;70(2):215-225.e6. doi:10.1016/j.annemergmed.2017.03.023 28641909

[32] Garthwaite C, Graves JA, Gross T, Karaca Z, Marone VR, Notowidigdo MJ. All Medicaid expansions are not created equal: the geography and targeting of the Affordable Care Act. Working paper 26289. National Bureau of Economic Research; October 14, 2019.

[33] Sommers BD, Simon K. Health insurance and emergency department use—a complex relationship. N Engl J Med. 2017;376(18):1708-1711. doi:10.1056/NEJMp1614378 28467870

[34] Friedman AB. The uncertain economics of insurance enabling more emergency department visits. Ann Emerg Med. 2017;70(2):226-228. doi:10.1016/j.annemergmed.2017.04.022 28641908

[35] Agency for Healthcare Research and Quality. Healthcare Cost and Utilization Project. Overview of the State Emergency Department Databases. September 2021. Accessed August 19, 2021. https://www.hcup-us.ahrq.gov/seddoverview.jsp

[36] Centers for Disease Control and Prevention. National Center for Health Statistics. Emergency department visits. Accessed April 2, 2022. https://www.cdc.gov/nchs/fastats/emergency-department.htm

[37] Kaiser Family Foundation. State health facts: demographics and the economy. Accessed October 1, 2021. https://www.kff.org/state-category/demographics-and-the-economy/population/

[38] Billings J, Parikh N, Mijanovich T. Emergency department use in New York City: a substitute for primary care? Issue Brief (Commonw Fund). 2000;(433):1-5. 11665698

[39] Status of state Medicaid expansion decisions: interactive map. Kaiser Family Foundation. April 26, 2022. Accessed January 27, 2021. https://www.kff.org/medicaid/issue-brief/status-of-state-medicaid-expansion-decisions-interactive-map/

[40] Giannouchos TV, Kum HC, Foster MJ, Ohsfeldt RL. Characteristics and predictors of adult frequent emergency department users in the United States: a systematic literature review. J Eval Clin Pract. 2019;25(3):420-433. doi:10.1111/jep.13137 31044484

[41] United States Bureau of Labor Statistics. TED: The Economics Daily. Accessed November 7, 2021. https://www.bls.gov/opub/ted/

[42] Wing C, Simon K, Bello-Gomez RA. Designing difference in difference studies: best practices for public health policy research. Annu Rev Public Health. 2018;39:453-469. doi:10.1146/annurev-publhealth-040617-013507 29328877

[43] Sommers BD, Gunja MZ, Finegold K, Musco T. Changes in self-reported insurance coverage, access to care, and health under the Affordable Care Act. JAMA. 2015;314(4):366-374. doi:10.1001/jama.2015.8421 26219054

[44] Agency for Healthcare Research and Quality. HCUP Fast stats—state trends in emergency department visits by payer. Accessed April 3, 2022. https://www.hcup-us.ahrq.gov/faststats/statepayer/statesED.jsp

[45] Tipirneni R, Rhodes KV, Hayward RA, Lichtenstein RL, Reamer EN, Davis MM. Primary care appointment availability for new Medicaid patients increased after Medicaid expansion in Michigan. Health Aff (Millwood). 2015;34(8):1399-1406. doi:10.1377/hlthaff.2014.1425 26202057

[46] Polsky D, Richards M, Basseyn S, . Appointment availability after increases in Medicaid payments for primary care. N Engl J Med. 2015;372(6):537-545. doi:10.1056/NEJMsa1413299 25607243

[47] Zuckerman S, Skopec L, Epstein M. Medicaid Physician Fees After the ACA Primary Care Fee Bump. Urban Institute; 2017.

[48] Neprash HT, Zink A, Sheridan B, Hempstead K. The effect of Medicaid expansion on Medicaid participation, payer mix, and labor supply in primary care. J Health Econ. 2021;80:102541. doi:10.1016/j.jhealeco.2021.102541 34700139

[49] Lowthian JA, Smith C, Stoelwinder JU, Smit DV, McNeil JJ, Cameron PA. Why older patients of lower clinical urgency choose to attend the emergency department. Intern Med J. 2013;43(1):59-65. doi:10.1111/j.1445-5994.2012.02842.x 22646852

[50] Giannouchos TV, Biskupiak J, Moss MJ, Brixner D, Andreyeva E, Ukert B. Trends in outpatient emergency department visits during the COVID-19 pandemic at a large, urban, academic hospital system. Am J Emerg Med. 2021;40:20-26. doi:10.1016/j.ajem.2020.12.009 33338676 PMC7725055

[51] Macinko J, Starfield B, Shi L. Quantifying the health benefits of primary care physician supply in the United States. Int J Health Serv. 2007;37(1):111-126. doi:10.2190/3431-G6T7-37M8-P224 17436988

[52] Wise-Harris D, Pauly D, Kahan D, Tan de Bibiana J, Hwang SW, Stergiopoulos V. “Hospital was the only option”: experiences of frequent emergency department users in mental health. Adm Policy Ment Health. 2017;44(3):405-412. doi:10.1007/s10488-016-0728-3 26961781

[53] Kirby S, Wooten W, Spanier AJ. Pediatric primary care relationships and non-urgent emergency department use in children. Acad Pediatr. 2021;21(5):900-906. doi:10.1016/j.acap.2021.03.019 33813066 PMC8263464

[54] Morganti KG, Bauhoff S, Blanchard JC, . The evolving role of emergency departments in the United States. Rand Health Q. 2013;3(2):3. 28083290 10.7249/rhq-03-02-03 PMC4945168

[55] Maxwell J, Bourgoin A, Lindenfeld Z. Battling the mental health crisis among the underserved through state Medicaid reforms. Health Affairs Blog. 2020;2020(Feb):346125. doi:10.1377/hblog20200205

[56] Weiss AJ, Barrett ML, Heslin KC, Stocks C. Trends in emergency department visits involving mental and substance use disorders, 2006–2013. HCUP Statistical Brief #216. Agency for Healthcare Research and Quality. December 2016. Accessed October 25, 2021. https://www.hcup-us.ahrq.gov/reports/statbriefs/sb216-Mental-Substance-Use-Disorder-ED-Visit-Trends.pdf 28121114

[57] Olfson M, Wall M, Barry CL, Mauro C, Mojtabai R. Impact of Medicaid expansion on coverage and treatment of low-income adults with substance use disorders. Health Aff (Millwood). 2018;37(8):1208-1215. doi:10.1377/hlthaff.2018.0124 30080455 PMC6190698

[58] Grogan CM, Andrews C, Abraham A, . Survey highlights differences in Medicaid coverage for substance use treatment and opioid use disorder medications. Health Aff (Millwood). 2016;35(12):2289-2296. doi:10.1377/hlthaff.2016.0623 27920318 PMC5304419

[59] Cummings JR, Wen H, Ko M, Druss BG. Race/ethnicity and geographic access to Medicaid substance use disorder treatment facilities in the United States. JAMA Psychiatry. 2014;71(2):190-196. doi:10.1001/jamapsychiatry.2013.3575 24369387 PMC4039494

[60] Patel SY, Mehrotra A, Huskamp HA, Uscher-Pines L, Ganguli I, Barnett ML. Trends in outpatient care delivery and telemedicine during the COVID-19 pandemic in the US. JAMA Intern Med. 2021;181(3):388-391. doi:10.1001/jamainternmed.2020.5928 33196765 PMC7670397

[61] Sommers BD. Health insurance coverage: what comes after the ACA? an examination of the major gaps in health insurance coverage and access to care that remain ten years after the Affordable Care Act. Health Aff (Millwood). 2020;39(3):502-508. doi:10.1377/hlthaff.2019.01416 32119630

[62] Sandberg SF, Erikson C, Owen R, . Hennepin Health: a safety-net accountable care organization for the expanded Medicaid population. Health Aff (Millwood). 2014;33(11):1975-1984. doi:10.1377/hlthaff.2014.0648 25367993

