# Supplementary material for: Association of Medicaid Expansion With Emergency Department Visits by Medical Urgency
Source: JAMA Netw Open. 2022 Jun 14;5(6):e2216913. doi: 10.1001/jamanetworkopen.2022.16913 (PMC9198732; doi:10.1001/jamanetworkopen.2022.16913)
Supplement: Supplement. — eFigure. Total Annual Number of Emergency Department Visits Across States From 2011 to 2017 eTable 1. Indirect Test of the Parallel Trends Assumption in the Pre-ACA Implementation Period eTable 2. Difference-in-Differences Regression Analyses Estimating Postexpansion Years Separately: Total ED Visits per 1000 Population and Stratified by Medical Urgency eTable 3. Difference-in-Differences Regression Analyses: Total ED Visits per 1000 Population and Stratified by Medical Urgency Between Florida and New York [file jamanetwopen-e2216913-s001.pdf]

Supplementary Online Content

Giannouchos TV, Ukert B, Andrews C. Association of Medicaid expansion with emergency department visits by medical urgency. *JAMA Netw Open*. 2022;5(6):e2216913. doi:10.1001/jamanetworkopen.2022.16913

eFigure. Total Annual Number of Emergency Department Visits Across States From 2011 to 2017

eTable 1. Indirect Test of the Parallel Trends Assumption in the Pre-ACA Implementation Period

eTable 2. Difference-in-Differences Regression Analyses Estimating Postexpansion Years Separately: Total ED Visits per 1000 population and Stratified by Medical Urgency

eTable 3. Difference-in-Differences Regression Analyses: Total ED Visits per 1000 Population and Stratified by Medical Urgency Between Florida and New York

This supplementary material has been provided by the authors to give readers additional information about their work.

© 2022 Giannouchos TV et al. *JAMA Network Open.*

eFigure : Total annual number of emergency department visits across states from 2011 to 2017

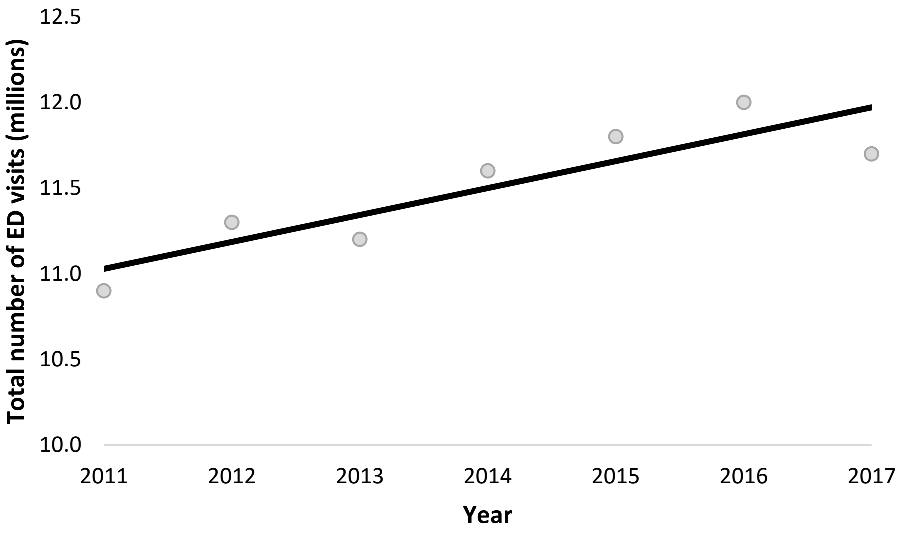

Trend in overall treat and release ED visits over time in four states.

© 2022 Giannouchos TV et al. *JAMA Network Open.*

eTable 1: Indirect Test of the Parallel Trends Assumption in the pre-ACA implementation period

	Total		Injuries / Not Preventable		Not Emergent		Preventable / Primary Care Treatable		Mental health / Substance Use Disorders	
	Coef.	*P*	Coef.	*P*	Coef.	*P*	Coef.	*P*	Coef.	*P*
2011/2	0.828	.80	-0.253	.85	0.261	.77	0.651	.45	0.096	.62
2011/3	1.917	.58	0.776	.58	0.316	.74	0.290	.75	0.240	.26
2011/4	0.708	.81	-0.221	.85	-0.011	.99	0.586	.50	0.139	.42
2012/1	-0.693	.82	-0.646	.55	-0.041	.96	-0.075	.93	0.008	.97
2012/2	-0.892	.77	-0.289	.82	-0.395	.64	-0.374	.61	0.060	.77
2012/3	0.906	.77	0.685	.60	-0.052	.95	-0.233	.77	0.132	.58
2012/4	-2.295	.44	-0.607	.57	-0.911	.29	-0.729	.44	-0.010	.96
2013/1	-1.469	.55	-0.271	.77	-0.690	.38	-0.694	.37	0.084	.67
2013/2	-0.712	.78	-0.061	.95	-0.632	.40	-0.282	.68	0.114	.59
2013/3	-0.581	.83	0.277	.80	-0.567	.48	-0.497	.47	0.084	.74
2013/4	-3.641	.14	-0.642	.50	-1.368	.09	-1.345	.10	-0.089	.66

The above table displays the coefficients and the p-values of the interaction term of year-quarter with the expansion status indicator in the pre-ACA period, with the first quarter in 2011 as the reference group. Data is limited to 2011-2013.

© 2022 Giannouchos TV et al. *JAMA Network Open.*

eTable 2: Difference-in-differences regression analyses estimating postexpansion years separately: total ED visits per 1000 population and stratified by medical urgency

No. of ED visits per 1,000 population	Adjusted Difference-in-Differences (95% CI)	*P*
**Total**		
2014	-3.9 (-6.7 - -1.1)	.007
2015	-4.7 (-8.0 - -1.4)	.006
2016	-6.9 (-9.9 - -4.0)	<.001
2017	-9.8 (-13.4 – -6.1)	<.001
**Injuries / Not Preventable**		
2014	-1.1 (-2.8 – 0.5)	.18
2015	-1.8 (-4.0 – 0.3)	.10
2016	-2.6 (-4.3 - -0.8)	.004
2017	-3.3 (-5.6 - -1.1)	.004
**Not Emergent**		
2014	-1.1 (-1.8 - -0.5)	.001
2015	-0.9 (-1.9 – 0.05)	.06
2016	-1.3 (-2.0 - -0.5)	.002
2017	-1.7 (-2.6 - -0.9)	<.001
**Primary Care Treatable**		
2014	-0.9 (-1.4 - -0.5)	<.001
2015	-0.9 (-1.5 - -0.2)	.01
2016	-1.4 (-2.1 - -0.6)	.001
2017	-1.9 (-2.7 - -1.1)	<.001
**Potentially Preventable**		
2014	-0.2 (-0.4 - -0.1)	.04
2015	-0.2 (-0.5 – 0.0)	.12
2016	-0.4 (-0.7 - -0.1)	.01
2017	-0.5 (-0.9 - -0.2)	.002
**Mental health / Substance Use Disorders**		
2014	-0.1 (-0.3 – 0.2)	.62
2015	-0.1 (-0.4 – 0.2)	.42
2016	-0.3 (-0.5 – 0.0)	.04
2017	-0.3 (-0.6 – 0.0)	.06

The analysis contained 112 state-year-quarters, 28 for each State. Of those, 48 state-year-quarters correspond to the pre-Medicaid expansion years (2011-2013). Results show adjusted differences-in-differences weighted estimates estimating post-expansion years separately for two expansion states (Massachusetts and New York) versus two non-expansion states (Florida and Georgia). Classification of ED visits by medical urgency was conducted using the New York University ED algorithm. Adjusted analyses controlled for age, gender, race/ethnicity, poverty levels, and unemployment. No: Number; CI: Confidence Intervals

© 2022 Giannouchos TV et al. *JAMA Network Open.*

eTable 3: Difference-in-differences regression analyses: total ED visits per 1000 population and stratified by medical urgency between Florida and New York

	FL	NY	Difference	Adjusted DiD	95% CI	*P*
**OVERALL**						
**Total**				-3.6	(-6.8 – -0.4)	.03
Before Medicaid expansion	50.8	50.6	-0.2			
After Medicaid expansion	54.9	50.0	-4.9			
**Injuries / Not Preventable**				-1.4	(-3.3 – 0.5)	.15
Before Medicaid expansion	16.6	16.8	0.2			
After Medicaid expansion	16.8	15.4	-1.4			
**Not Emergent**				-0.8	(-1.6 – -0.1)	.03
Before Medicaid expansion	12.1	11.8	-0.3			
After Medicaid expansion	12.5	11.1	-1.4			
**Primary Care Treatable**				-0.6	(-1.1 – -0.1)	.02
Before Medicaid expansion	11.8	10.3	-1.5			
After Medicaid expansion	12.7	10.3	-2.4			
**Potentially Preventable**				-0.2	(-0.4 – -0.1)	.03
Before Medicaid expansion	2.7	2.7	0.0			
After Medicaid expansion	2.9	2.6	-0.3			
**Mental health / Substance Use Disorders**				0.0	(-0.2 – 0.2)	.91
Before Medicaid expansion	1.5	3.1	1.6			
After Medicaid expansion	1.7	3.2	1.5			

The analysis contained 56 state-year-quarters, 28 for each State. Of those, 24 state-year-quarters correspond to the pre-Medicaid expansion years (2011-2013). Results show differences-in-differences weighted estimates for New York versus Florida. Classification of ED visits by medical urgency was conducted using the New York University ED algorithm. Adjusted analyses controlled for age, gender, race/ethnicity, poverty levels, and unemployment. No: Number; CI: Confidence Intervals

© 2022 Giannouchos TV et al. *JAMA Network Open.*
